# Prognostic value of semi-quantitative bacteruria counts in the diagnosis of group B streptococcus urinary tract infection: a 4-year retrospective study in adult patients

**DOI:** 10.1186/1471-2334-12-273

**Published:** 2012-10-26

**Authors:** Chee K Tan, Kimberly B Ulett, Michael Steele, William H Benjamin, Glen C Ulett

**Affiliations:** 1 School of Medical Sciences, Centre for Medicine and Oral Health, Griffith University, Gold Coast Campus, Queensland, 4222, Australia; 2 Department of Medicine, University of Alabama at Birmingham, Birmingham, AL, 35294, USA; 3 Bond University, Gold Coast, Queensland, 4229, Australia; 4 Department of Microbiology, University of Alabama at Birmingham, Birmingham, AL, 35294, USA; 5 Department of Pathology, University of Alabama at Birmingham, Birmingham, AL, 35294, USA; 6 Present address: Royal Brisbane and Women’s Hospital Department of Medicine, Bowen Bridge Road, Herston, Queensland, 4029, Australia

**Keywords:** Urinary tract infection, Cystitis, Bacteruria, *Streptococcus agalactiae*, Asymptomatic bacteruria, Urinalysis, Uropathogen

## Abstract

**Background:**

Semi-quantitative bacteruria counts (s-QBC) are important in the diagnosis of urinary tract infection (UTI) due to most uropathogens. The prognostic value of s-QBC for diagnosis of UTI due to group B streptococcus (GBS) is unknown. In this study, we assessed the value of s-QBC for differentiating acute GBS UTI from asymptomatic bacteruria (ABU), independent of other potential prognostic indicators.

**Methods:**

Medical record review and urinalysis (UA) values for 1593 patients who had urinary GBS isolated (10^3^ to ≥10^5^ CFU/ml) during a four-year period were analyzed using binary logistic regression to determine the predictive values of s-QBC, age, and gender for infection category (acute UTI, ABU) based on the clinical diagnosis.

**Results:**

s-QBC alone had a strong predictive value for infection category but only for ABU. Multivariate logistic regression showed similar predictive power of s-QBC for infection category using age as a co-predictor, which was also independently associated with infection category. Typical s-QBC cut-off values that are commonly used in diagnostic settings had no significant power in predicting infection category. Among other UA measures, proteinuria and hematuria were significantly associated with acute infection.

**Conclusions:**

Together, these data show that s-QBC is not useful in the differential diagnosis of GBS UTI. Among the patients in this study, age was an equally effective prognostic indicator compared to s-QBC for identifying high- and low-risk patients for acute GBS UTI. Collectively, these findings indicate that age-based associations may be equally as useful as s-QBC for predicting infection category in the setting of adult patients with GBS-positive urine cultures.

## Background

Bacterial urinary tract infections (UTIs) are among the most common infectious diseases of humans. One in three women will contract a UTI in their lifetime [1], and approximately 3% of these individuals will experience more than one infection per year [2,3]. UTIs contribute to approximately 60 million hospital visits per year with substantial associated costs to the healthcare industry [2-5]. Over 80% of UTIs are caused by *Escherichia coli* and other Gram-negative bacteria including *Pseudomonas* spp*.*, *Klebsiella* spp*.*, and *Acinetobacter* spp. many of which are increasingly being associated with antibiotic resistance [6-9].

Gram-positive bacteria including enterococci, staphylococci, and streptococci are also important uropathogens. These organisms are less prevalent compared to Enterobacteriaceae but constitute major contributors to the global burden of UTI [6,7]. *Streptococcus agalactiae*, also known as group B streptococcus (GBS), emerged as an important cause of acute UTI in adults in the 1990’s, and various forms of GBS infection of the urinary tract have been described in subsequent studies [10-14]. While generally associated with maternal cervicovaginal colonization and neonatal disease, GBS also frequently causes serious infections including acute UTI in older persons and those with chronic medical illness [15,16]. The spectrum of GBS UTI encompasses asymptomatic bacteruria (ABU), cystitis, pyelonephritis, and urosepsis. Clinically, acute GBS UTI is indistinguishable from acute UTI caused by other uropathogenic bacteria, and diagnosis is made difficult by high rates of sample contamination and asymptomatic infection [14,17]. GBS is cultured from urine in approximately 2% of all cases of clinically suspected UTI [10,14,18,19]. In addition, urinary GBS complicates up to 7% of pregnancies and contributes up to 10% of cases of pyelonephritis in pregnancy [17,19-21]. GBS ABU is considered particularly important during pregnancy due to the risk of vertical transmission of the organism, which mandates antibiotic therapy [22]. However, the relative significance of ABU due to GBS in pregnancy compared to that due to other organisms remains unclear [23,24].

Diagnostic strategies and use of the recommended treatment guidelines for bacterial UTI varies substantially between clinicians [25-28]. A combination of symptoms in adults is considered predictive with a high degree of probability for acute infection [29,30], however, there can be difficulties in applying general rules to specific patient populations such as the elderly [28]. In the laboratory setting, semi-quantitative bacteruria counts (s-QBC) are routinely used as a diagnostic criterion for individuals suspected of having a UTI. Typically, s-QBC ranging between 10^3^ and ≥10^5^ CFU/ml are used for laboratory-based prediction of acute uncomplicated UTI in symptomatic patients. However, there exists debate on the interpretation of s-QBC for diagnostic criteria, and revisions of the traditionally accepted 10^5^ CFU/ml cut-off value for acute UTI have emerged. Several studies have suggested that this s-QBC cut-off may be inappropriate for some clinical conditions, and modified lower and higher cut-off values ranging from 10^3^ to ≥10^6^ CFU/ml have been proposed [30-33]. In addition, many laboratories continue to disregard s-QBC of <10^5^ CFU/ml for Gram-positive bacterial uropathogens, and there remains debate regarding the prognostic value of s-QBC of <10^4^ CFU/ml for diagnosis of UTI due to different organisms [34].

The aim of this study was to investigate the prognostic value of s-QBC in the diagnosis of acute GBS UTI in the setting of adult patients with urine cultures positive for this organism. We retrospectively analyzed data for the largest cohort of patients described to date in order to examine whether s-QBC is useful as a predictor of acute GBS UTI independent of other variables. The findings show that s-QBC is not useful in the prediction of infection category, and suggest that age-based associations may be equally as valuable compared to s-QBC for the differential diagnosis of acute UTI and ABU due to GBS. This study represents the first analysis of the role of s-QBC in the diagnosis of GBS UTI, and despite some limitations, the findings reported here provide useful new insight into the interpretation of s-QBC values for GBS UTI.

## Methods

### Study design

This study was conducted using clinical and laboratory data from a single-center analysis of GBS UTI in patients at University of Alabama at Birmingham (UAB) Hospital conducted between August-2007 and August-2008 [14]. Additional patients who attended the UAB Hospital or surrounding clinics in the Birmingham area in the subsequent 36-month period, and who tested positive for urinary GBS, were also included in this study. Consequently, the data analyzed here represents all patients who presented with positive urine cultures for GBS during the 48-month period between August-2007 and July-2011. The study was performed with approval from and in accordance with the ethical standards of the UAB committee on human experimentation and the Helsinki Declaration (approval X070722011). Ethical approval was also sought and granted by the Griffith University human research ethics committee (approval MSC/02/11/HREC). Written informed consent from participants for the publication of images and patient data was obtained according to the approvals from the UAB committee on human experimentation and the Griffith University human research ethics committee.

### Study participants

The study subjects were adult patients (>18 yrs) encountered at UAB Hospital and surrounding clinics who underwent clinical and microbiological assessment for UTI because of symptoms indicating infection or as part of routine patient screening. Urine samples were obtained as clean-catch voided or catheterized samples from all patients who underwent assessment for UTI during the study period. This included inpatients, patients that were evaluated in the emergency department, and patients from various University Hospital outpatient clinics. In cases where GBS was cultured from urine (any count, single organism and mixed cultures) the medical records for each patient were reviewed for presenting symptoms at the time of sample collection and demographic data were recorded. Potential prognostic indicators were collected by medical record review to encompass, on average, the three-week period either side of the date when the sample was collected. A provisional diagnosis of acute GBS UTI was defined by the presence of single-organism GBS bacteruria (any count; limits of detection, 10^3^ to ≥10^5^ CFU/ml) with ≥1 symptom that included dysuria, increased urinary frequency and/or urgency, fever >38°C, flank pain and/or lumbar tenderness. All GBS isolates cultured from urine were identified by typical colony morphology on BBL trypticase soy agar with 5% sheep blood (Becton, Dickinson and Company, Franklin Lakes, NJ), tested for catalase, and grouped using the Remel PathoDx latex agglutination kit (Thermo Fisher Scientific, Lenexa, KS).

In cases where urinalysis (UA) was undertaken as part of the diagnosis, acute UTI was confirmed on the basis of positive urinary leukocyte esterase and significant pyuria (≥10^7^ white blood cells/L; non-spun). Thus, the case definition for acute GBS UTI in this study was symptomatic patients with a s-QBC of any level and UA findings consistent with (C/W) acute infection. This definition was taken as appropriate since acute UTI has been described in patients with s-QBC as low as 10^2^ CFU/ml in a prior study [14]. Exclusion criteria were the isolation of multiple organisms from urine, or incomplete medical records. GBS ABU was defined as single-organism bacteruria of any count, in the absence of UTI symptoms. This definition was chosen as rationally inclusive since GBS s-QBC may vary considerably in individual patients over several hours [14]. Additional inclusion criteria for GBS ABU were negative UA laboratory values for leukocyte esterase and pyuria, where UA had been undertaken. All study subjects who satisfied these inclusion criteria were allocated to one of two infection category groups: acute GBS UTI, or ABU. This allowed analyses of relationships between GBS UTI infection category, s-QBC, age, gender, and other UA measures.

### Data analysis

In the first stage of the statistical analysis, to determine the overall predictive ability of s-QBC for differentiation of acute GBS UTI from ABU, a number of univariate logistic regression models were applied. These models examined the independent effects of s-QBC, age, and gender on infection category based on the clinical diagnosis. The variables included were age, gender, s-QBC, and infection category (acute UTI and ABU). Specific interactions between potential prognostic factors were subsequently evaluated using a multivariate logistic regression model in which all variables were included to determine whether there existed any significant relationships between each of the individual potential prognostic factors in the prediction of infection category. The mean age and s-QBC for patients in different infection categories were compared using an Independent Sample t-test.

In the second stage of data analysis, we investigated a subset of study subjects for whom UA testing had been undertaken as part of the diagnosis. For all of the cases that had UA performed, we analyzed the potential relationships between s-QBC and other laboratory variables using a multivariate logistic regression model to determine any significant interactions in the prediction of infection category. The additional variables included were UA measures of specific gravity, pH, proteinuria, hematuria, glucose, ketones, and bilirubin. All statistical analyses were carried out using IBM SPSS Statistics software (Version 19.0). A level of significance of p < 0.05 was selected for all of the analyses.

## Results

### Study subjects

A total of 1593 patients had GBS isolated from urine during the study period. Within this cohort, 573 patients satisfied the inclusion criteria and were assigned to either one of the two infection category groups, acute GBS UTI or ABU. The majority of the 1020 residual subjects were excluded due to the isolation of multiple organisms, or in some cases, due to unobtainable medical records. Gender and age data for the 573 study participants are shown in Table 1, with mean s-QBC values for patients divided according to infection category and gender. There were 497 women and 76 men among the 573 study subjects (ratio 6.5:1), with average ages of 42.4 and 53.1 years, respectively (Table 1). There were 147 patients diagnosed with acute GBS UTI, and 426 patients with ABU. The mean age of patients with acute UTI was 51.1 years, which was higher than patients with ABU (41.3 years; p<0.001).

**Table 1:** Age, gender, and semi-quantitative bacteruria count (s-QBC) data for 573 patients with acute GBS UTI or ABU at University of Alabama at Birmingham Hospital between August 2007 - July 2011

	**All Cases In Study (n=573)**	**Acute UTI Cases (n=147)**	**ABU Cases (n=426)**
**Females**	497 (86.75%)	130 (88.44%)	367 (86.15%)
Mean age, and range	42.4, 18-93	50.8, 19-93 (p<0.001)^†^	36.7, 18-88
Mean s-QBC (CFU/mL)	51,145 ± 1,767	64,369 ± 3,169 (p<0.001)^†^	46,460 ± 1,181
**Males**	76 (13.25%)	17 (11.56%)	59 (13.85%)
Mean age, and range	53.1, 20-91	58.7, 25-91 (p=0.151)^#^	51.5, 20-80
Mean s-QBC (CFU/mL)	48,697 ± 4,580	61,000 ± 9,818 (p=0.165)^#^	45,153 ± 5,129
**Combined**	573 (100%)	147 (100%)	426 (100%)
Mean age, and range	43.8, 18-93	51.1, 19-93 (p<0.001)^*^	41.3, 18-88
Mean s-QBC (CFU/mL)	50,820 ± 1,557	63,980 ± 3,013 (p≤0.001)^*^	46,279 ± 1,768

Statistical Comparisons: ^†^All Female Cases C/W acute UTI compared to All Female Cases C/W ABU. ^#^All Male Cases C/W acute UTI compared to All Male Cases C/W ABU. ^*^All Cases C/W acute UTI compared to All Cases C/W ABU.

### Bacteruria counts

The mean s-QBC for patients with acute GBS UTI was 63,980 ± 3,013 CFU/ml, which was significantly higher than patients with ABU (46,279 ± 1,768 CFU/ml; p≤0.001) (Table 1). These data, together with the data described above, imply a relationship between infection category and s-QBC, as well as infection category and age for GBS UTI, in which patients with acute GBS UTI are older, with higher s-QBC values, compared to patients with ABU.

The differences in mean age and s-QBC values between patients in different infection category groups related to women more so than men. Comparisons of mean ages, divided according to gender, showed a significantly higher mean age of women with acute UTI (50.8 years) compared to women with ABU (39.7 years; p<0.001). While the mean age of men with acute UTI was also higher (58.7 years) compared to men with ABU (51.5 years), this difference was not statistically significant (p=0.151). Collectively, these data show that acute GBS UTI is associated with increased age in women, but there is no significant difference in the mean age of men with acute GBS UTI versus ABU.

Mean s-QBC values for women with acute UTI (64,369 ± 3,169 CFU/ml) were significantly higher than women with ABU (46,460 ± 1,181 CFU/ml) (p<0.001). This was not true for men (Table 1). Overall, patients with acute UTI were also more likely to be women than men (OR 7.7, 95% CI, 4.2-13.9). Taken together, these data show that the differences in mean age and s-QBC values between individuals with acute UTI and ABU in the study cohort were more strongly related to women than men.

### Potential prognostic indicators

Initial univariate logistic regression analysis revealed a significant relationship between s-QBC and infection category among all study subjects (Table 2). This was consistent with the significantly higher mean s-QBC value in acute UTI patients compared to that in individuals with ABU (Table 1). However, this relationship between s-QBC and infection category (p<0.001) was related to ABU cases only; i.e. s-QBC predicted 426/426 ABU cases correctly, but none of the 147 acute UTI cases were predicted. As a result of this, we tested three commonly applied s-QBC cut-off values in this model (10^3^, 10^4^, 10^5^ CFU/ml); however, none of these increased the predictive power of s-QBC for infection category because the percentage of cases that were correctly classifed remained at 74.3% (Table 2). These data show that while patients with acute GBS UTI have significantly higher mean s-QBC values than those with ABU, s-QBC alone is not useful as a predictor of infection category. The univariate model also showed a significant relationship between infection category and age, which classified 74.7% of subjects correctly into infection category with significant predictive power (p=0.025). In this analysis, age correctly predicted 3/147 patients with acute GBS UTI and 425/426 patients with ABU, compared to 0/147, and 426/426 predicted by s-QBC. Thus, age alone was equally as useful as a predictor of UTI compared to s-QBC in the overall study cohort as analysed using this model.

**Table 2:** Prognostic values of age and semi-quantitative bacteruria count (s-QBC) for differentiating cases of acute GBS UTI and ABU

**Testing Model**^**†**^	s-QBC^*^	Age^*^	**Constant**	**% Correct**^**#**^
**Univariate (s-QBC)**				
Continuous s-QBC	1.13x10^-5^	NA	-1.776	74.3%
C/O: 10^3^, 10^4^ or 10^5^	0.92-1.10	NA	-1.13-2.02	74.3%
**Univariate (Age)**				
Continuous Age	NA	0.025	-2.24	74.7%
**Multivariate**				
Continuous s-QBC	1.04x10^-5^	0.021	-2.59	74.7%
C/O: 10^3^, 10^4^ or 10^5^	0.76-0.87	0.022-4	-2.32-2.97	74.7%

^†^ C/O: Cut-off value applied for s-QBC in CFU/ml. ^*^ Values in columns represent p values. ^#^ Correct classification of cases as acute GBS UTI or ABU infection category as determined by the testing model.

We also analyzed potential relationships between s-QBC, age, and gender for the prediction of infection category using a multivariate model with the results shown in Table 2. Here, the interactions between s-QBC and age did not increase predictive power for infection category, and similar predictive abilities were observed (p=0.021 to 0.024) regardless of whether s-QBC was analyzed as a continuous variable, or as groups using nominal cut-off values. Gender did not alter the predictive ability of s-QBC when included in the multivariate model (not shown). Thus, interactions between s-QBC and age do not increase the predictive power for GBS UTI infection category compared to either variable alone.

We next analyzed whether an age-based cut-off might increase the predictive power of s-QBC for infection category. For this, we initially applied a cut-off of 40 years, which we based on the finding that individuals aged 40 years or older in this study were at significantly increased risk for acute GBS UTI compared to ABU (OR 1.7, 95% CI, 1.3-1.9). However, we found no increase in the predictive power of s-QBC for acute UTI using this model. Further analyses of alternative age cut-offs revealed similar increased risks (35 years: OR 1.5, 95% CI, 1.3-1.7; 45 years: OR 1.6, 95% CI, 1.3-1.9; 50 years: OR 1.5, 95% CI, 1.2-1.8), but no significant effect towards predictive power of s-QBC. Collectively, these data show that age is a useful prognostic indicator for infection category in GBS UTI, however, it does not enhance the predictive power of s-QBC in the differential diagnosis of these infections.

In the second stage of the statistical analysis we investigated a subset of 245 subjects for whom UA testing had been undertaken as part of the diagnosis. The laboratory findings from UA for these 245 patients, divided according to infection category, are shown in Table 3. In a multivariate model, there were no significant interactions between any of the variables; i.e. the predictive power of s-QBC was the same whether or not the UA variables were included in the analysis. Separate univariate models identified significant associations between infection category and proteinuria (p=0.03), and infection category and haematuria (p=0.025; Table 3). For other UA measures, such as glucose and ketones, differences between mean values of infection category groups were not significantly different.

**Table 3:** Urinalysis data for adult patients diagnosed with acute GBS UTI or GBS ABU at University of Alabama at Birmingham Hospital during the study period

	**UA for All Cases (n=245)**	**UA for Acute UTI Cases (n=75)**	**UA for ABU Cases (n=170)**	**P Value**
**Mean s-QBC (CFU/mL)**	59,792 ± 2,350	73,347 ± 3,823	53,812 ± 2,825	<0.001
**Specific Gravity**	1.016 ± 0.008	1.016 ± 0.008	1.005 ± 0.085	0.354
**pH**	6.36 ± 0.8	6.41 ± 0.1	6.33 ± 0.1	0.522
**Proteinuria**	0.77 ± 1.10 (118, 48.1%)	0.57 ± 0.09 (35, 46.6%)	0.86 ± 0.09 (83, 48.8%)	0.030^†^
**Glucose**	0.26 ± 0.70 (46, 19.2%)	0.21 ± 0.08 (8, 10.1%)	0.29 ± 0.05 (38, 22.4%)	0.461
**Ketones**	0.16 ± 0.51 (33, 13.5%)	0.13 ± 0.04 (11, 14.7%)	0.17 ± 0.04 (22, 12.9%)	0.564
**Bilirubin**	0.01 ± 0.11 (3, 0.01%)	0.01 ± 0.01 (1, 1.3%)	0.01 ± 0.01 (2, 1.1%)	0.918
**Hematuria**	0.68 ± 0.94 (119, 48.6%)	0.90 ± 0.11 (45, 60.0%)	0.59 ± 0.07 (74, 43.5%)	0.025^†^

Statistical Comparisons: † All Cases with UA C/W Acute GBS UTI compared to All Cases with UA C/W GBS ABU. Numbers in parentheses in columns represent the n of subjects who exhibited a positive result for this test, followed by this number expressed as a percentage of the total n of cases in the group.

## Discussion

The principle finding of this study is that in adult patients with acute GBS UTI, while mean s-QBC values are higher than in patients with ABU, these measures are not useful in distinguishing between these conditions. These findings are noteworthy given the widespread application of s-QBC values for identifying acute UTI in patients who are culture-positive for various organisms, including GBS. The finding that s-QBC is not a significant prognostic indicator for acute GBS UTI has practical implications in terms of disregarding low s-QBC values in GBS-positive patients. Low-count s-QBC is sometimes associated with acute infection [14], and the data reported here suggest that interpreting low-count s-QBC in GBS-positive patients as merely transient flora, or contaminants is inappropriate. Thus, nominal s-QBC cut-off values commonly used such as ≥10^5^ CFU/ml and ≥10^4^ CFU/ml should be considered as insensitive for the diagnosis of acute GBS UTI. The data presented here suggest that a cut-off of ≥10^3^ CFU/ml might be appropriate (the limit of detection for most laboratories). This is consistent with recent ideas highlighting the value of lower s-QBC cut-offs in the setting of adult patients infected with other uropathogens [30,32-34].

Semi-quantitative urinary cultures have been reported in several studies in which over half of symptomatic patients exhibited s-QBC values lower than the traditional cut-off of 10^5^ CFU/ml often used for defining significant bacteruria [35,36]. Others have reported that a ≥10^2^ CFU/ml s-QBC criterion may be superior to a ≥10^5^ threshold [37,38]. In a report by Thomsen *et al.* that investigated penicillin treatment of patients with GBS s-QBC of any count, there was a reduction in poor pregnancy outcomes compared to a placebo control, also suggesting that s-QBC lower than the traditional cut-off of 10^5^ CFU/ml are clinically important [39]. These findings show that s-QBC values as low as 10^2^ CFU/ml are especially significant in pregnant women. In the broader context, women with low-grade bacteruria due to other uropathogens have been shown to respond to therapy [40] supporting the notion that low-grade s-QBC values are important for uropathogens in general.

It is important to highlight the limitations of the current study, and the possible implications of lowering the s-QBC cut-off value for interpreting GBS culture-positive patients in the diagnostic setting of UTI. Contamination of specimens in the current work could not be suitably addressed due to the retrospective nature of the study and the unfeasibility of repeat urine cultures and catheterized samples for all patients in this setting. Overall, the number of catheterized samples was low (~10%), and related to both acute UTI and ABU patients. We defined ABU as the isolation of urinary GBS in pure culture from a single sample, which is an approach consistent with prior studies [14,41]. Others have limited the definition of ABU to cases in which two- or three-consecutive samples from an individual are culture-positive for the same or similar organism [32,42,43]. Our goal was to incorporate the highest number of cases possible into our study, and its retrospective design ruled out repeat sampling. Thus, some of the asymptomatic women with urinary GBS in this study would represent cases of sample contamination, especially for low count samples, whilst others may reflect low-grade bacteruria. Despite this limitation, data related to symptomatic patients with low-grade s-QBC, and thus, the key finding of the study, remain valid. Future studies of subjects with repeat samples incorporating a higher proportion of catheterized patients would be useful to address this.

Contamination and recovery of multiple organisms occurs in approximately half of all urine specimens that are positive for GBS [14], and we excluded all such cases in the current study. In contaminated specimens, other organisms may overwhelm low numbers of GBS causing a large number of UTIs due to this organism to be missed. There were also many patients with high GBS s-QBC but much lower numbers of other organisms in the current study (also excluded), which probably indicates a real infection but is discounted in the diagnostic laboratory as a contaminated specimen due to reporting. Overall, this means that many true GBS UTI cases were probably missed in this study (only single-organism cultures were used per case definitions). In the broader context, such cases continue to be missed in microbiology laboratories due to routine reporting practices in the diagnostic setting. Finally, a high prevalence of low-grade GBS bacteruria [42] also means that revising down the s-QBC cut-off for GBS UTI would increase the rate of false positives. Despite these challenges, the data reported here show that a ≥10^5^ CFU/ml threshold is insensitive as a marker for acute GBS UTI, which provides important new practical information for the diagnostic laboratory.

This study also suggests that age is a useful prognostic indicator for predicting acute GBS UTI, perhaps equally so as compared to s-QBC. Despite this, age still only identified 3/147 acute GBS UTI patients in our patient cohort based on a logistic regression model. The age measure was confounded in our study because many of the urine specimens obtained from asymptomatic women (~25-30%) were primarily related to perinatal screening of pregnant women. Nonetheless, patients over the age of 40 years were shown to be at significantly higher risk for acute GBS UTI compared to younger patients, suggesting age may be useful as a surrogate risk marker. This finding, against the background of high false positive rates for acute GBS UTI, highlights the need for careful interpretation of GBS-positive urine cultures. Using age as a prognostic tool could help to identify high-risk patients and partly address the difficulties in interpreting s-QBC values for GBS. Gender was not a prognostic factor for discriminating infection category, which may be more relevant to older persons and nursing home residents as discussed elsewhere [44].

GBS infection in adults is often associated with diabetes, which is considered a risk factor for disease due to this organism [45]. The prevalence of ABU in diabetic individuals is higher compared to individuals without diabetes [46], and although diabetic patients are more likely to have acute UTI due to any uropathogen, diabetes was not identified as a risk factor for GBS UTI in a prior study [14]. This suggests that the association between diabetes and symptomatic UTI may not be as relevant for GBS. The diabetic status of all study subjects in the current work was not included in the medical record review. Interestingly, however, the proportion of individuals in the current study with ABU who exhibited a positive test result for elevated urinary glucose was more than double that of patients with acute GBS UTI (22.4% vs. 10.1% respectively, a significant difference according to Pearson Chi-Square analysis) hinting at a possible connection. The role of diabetes in GBS UTI and possible bias in the current dataset due to differential pregnancy rates in different patient groups will require further investigation. The association between bacteruria and diabetes in relation to microbial sugar metabolism also raises the possibility of growth of uropathogenic organisms in urine [47]. Growth fitness in urine has been shown for *E. coli*[48], but steps in the pathogenesis of GBS UTI have only recently been described [49-51], and a GBS isolate cultured from a patient with acute cystitis was unable to grow in urine [51]. Proteinuria in ABU patients in this study also implies that diabetes may be associated with GBS ABU. Comparing the proteinuria and hematuria data in ABU patients irrespective of esterase and pyuria UA findings (possible bias per the exclusion criteria) would also be of interest for further study.

## Conclusion

This study shows that s-QBC is not useful as a prognostic measure in the diagnosis of acute GBS UTI. Whilst many laboratories continue to disregard s-QBC values <10^5^ CFU/ml some are now using a threshold of ≥10^3^ CFU/ml for voided specimens containing uropathogenic bacteria to increase the sensitivity of detection. This would be appropriate for GBS. This study also demonstrates that age over 40 years is a useful surrogate marker for identifying high risk patients for acute GBS UTI. Further investigations of ways to improve basic diagnostic approaches for these important infections are now warranted.

## Abbreviations

GBS: Group B streptococcus; s-QBC: Semi-quantitative bacteruria count; UTI: Urinary tract infection; ABU: Asymptomatic bacteruria; UA: Urinalysis; CFU: Colony forming unit; OR: Odds ratio.

## Competing interests

The authors declare that they have no competing interests.

## Authors’ contributions

CKT participated in the study design and its coordination, helped perform the statistical analysis, and interpreted the data. KBU performed the medical record review, interpreted the data, and helped draft the manuscript. MS performed the statistical analysis and interpreted the data. WHB and GCU conceived of the study, participated in its design and coordination, managed the ethical considerations and obligations for the study, interpreted the data, and drafted the manuscript. All authors read and approved the final manuscript.

## Pre-publication history

The pre-publication history for this paper can be accessed here:

http://www.biomedcentral.com/1471-2334/12/273/prepub
